# Human Leukocyte Transcriptional Response to SARS-CoV-2 Infection

**DOI:** 10.6061/clinics/2020/e2078

**Published:** 2020-06-16

**Authors:** Sandra Elisabete Vieira, Silvia Yumi Bando, Gerhard da Paz Lauterbach, Carlos Alberto Moreira-Filho

**Affiliations:** I Departamento de Pediatria, Faculdade de Medicina FMUSP, Universidade de Sao Paulo, Sao Paulo, SP, BR.; II Divisao de Clinica Medica, Hospital das Clinicas HCFMUSP, Sao Paulo, SP, BR.

It is necessary to elucidate why in the present coronavirus disease-19 (COVID-19) global pandemic infection by SARS-CoV-2 affects children, adults, and elderly people differently. Epidemiological and clinical data have shown that most children who test positive for SARS-CoV-2 are either asymptomatic or develop mild symptoms (1-3). Several hypotheses have been suggested to explain these findings: differences in the immune system of adults and children (and between very young children, pre-schooling children, and adolescents); competition with other respiratory viruses in the respiratory mucosa and lungs influencing the viral load; and differences between adults and children in the expression of the ACE2 protein, the SARS-CoV-2 receptor (1). It was recently described that the hypomethylation and subsequent hyperexpression of the *ACE2* gene increased susceptibility for developing COVID-19 (4).

In severe COVID-19 cases, the viral and pulmonary phases are followed by a final hyperinflammatory phase, which can lead to severe acute respiratory distress syndrome (ARDS), often with a fatal outcome. Here, it should be noted that children are quite susceptible to H1N1-related ARDS (5) and that the 2003 coronavirus SARS pandemic affected patients of all ages (6). Therefore, despite the relatively small number of reported COVID-19 cases in children and the scarce information on these cases, it is not possible to assume that all pediatric COVID-19 cases will follow a mild course (7).

The study of the differences between children and adults with COVID-19 regarding the immune response and disease course represents a unique opportunity for developing new therapies (8), which is demanded (9) to avoid the collapse of health systems now and in the immediate post-pandemic period (2022-2024), as shown by recent epidemiological studies (10). Consequently, we decided to investigate the genomic basis of those differences through a comparative study of the transcriptional responses of human leukocytes to SARS-CoV-2 infection in children and adults, also focusing on the differences between oligosymptomatic and severe cases, as further described in the following paragraphs.

Severe COVID-19 cases are characterized by a “cytokine storm” (hypercytokinemia) that promotes hyperinflammation and ARDS (11,12), which is not observed in oligosymptomatic cases (13). The inflammatory responses in adults and children vary with age, with a progressive increase in inflammatory cytokines and neutrophil activity, which correlates with the augmented severity of ARDS in elderly people. Even in pediatric septic shock, the vast majority of genes with altered expression profiles are in neutrophils (69%) and monocytes (28%), and just a small minority are in lymphocytes (14). Therefore, it is quite probable that in circulating leukocytes distinct transcriptional modules (see below) are associated with different responses to SARS-CoV-2 in COVID-19 patients, thus allowing us to delineate adult and child responses and, in these two groups, the oligosymptomatic and severe case subgroups. The functional analysis of these transcriptional modules will allow, as commented on below, a better understanding of the pathogenic mechanisms triggered by SARS-CoV-2 and eventually, the identification of new therapeutic targets.

The availability of platforms for large scale gene expression analysis—mainly DNA microarrays and next generation sequencing (NGS)—has made it possible for immune response studies to migrate from a reductionist approach to one of systems biology (15), enabling a global perception of the molecular, cellular, and tissue events involved in the different types of immune response (16). Studies at this new global transcriptome scale have permitted, for instance, a better understanding of the innate and adaptative immune responses, of the defense mechanisms against different pathogens, and the evaluation of the responses to vaccination (17-21).

An initial major hurdle in global transcriptome studies was how to analyze and interpret the enormously large gene expression datasets obtained through DNA microarrays or NGS platforms. The development of statistical and computational tools for the analysis of gene co-expression networks helped to overcome this limitation (22,23). These tools are presently used for associating genes and gene expression profiles with biological processes and for finding potential therapeutic targets (24-25). Clustering techniques have been employed to find genes with similar expression patterns in multiple samples, thus identifying modules (26,27). Transcriptional modules often represent biological processes and can be phenotype specific (25). The functional enrichment among the genes within a module is widely used for disclosing its biological meaning (25). Moreover, it was found that in gene co-expression networks, the highly connected genes hold the whole transcriptional network together and are either associated with specific cellular processes or link different biological processes (23). Connectivity measures are currently used for the hierarchical categorization of genes in transcriptional modules highly correlated with at least one trait of interest (gender, age, disease features, etc.), helping to find genes that are highly significant for a certain trait or that link molecular pathways in a cell (25).

The development of mathematical and computational methods for analyzing modular transcriptional repertoires has been essential for unraveling the human immune defense mechanisms associated with good and bad responses to respiratory viruses (17,28-30). Our group, at the Department of Pediatrics, FMUSP, has tackled this approach for investigating the genomic mechanisms associated with the development, maturation, and decline of the immune system in health and disease (31-33). Recently, studying children under six months of age hospitalized with acute viral bronchiolitis, we were able to show that in peripheral blood mononuclear cells (PBMC) there are distinct transcriptional modules associated either with responses to syncytial respiratory virus (HRSV) or rhinovirus (HRV) (20). We also identified host-response molecular markers that could be used for etiopathogenic diagnosis. The finding of distinct transcriptional profiles associated with specific host responses to HRSV or HRV may contribute to unraveling the pathogenic mechanisms triggered by different respiratory viruses that are indistinguishable by clinical presentation, paving the way for new, specific therapeutic strategies.

The experimental approach first adopted for studying the PBMC response to HRSV and HRV is now being used in our laboratory to identify the transcriptional responses of human peripheral blood leukocytes to SARS-CoV-2 following respiratory tract infection. Relevant knowledge on this subject has been newly published. Transcriptome characteristics of the bronchoalveolar lavage fluid and peripheral PBMC of COVID-19 patients revealed distinct host inflammatory cytokine profiles and the association between COVID-19 pathogenesis and excessive cytokine release (34). Compared to other respiratory viruses, SARS-CoV-2 drives a lower antiviral transcriptional response—low IFN-I and IFN-III levels and elevated chemokine expression—in accordance with the pro-inflammatory disease state associated with COVID-19 (35). In a complementary line of work, COVID-19 patients were compared to recovered and healthy subjects through high dimensional cytometry, and the subsequent integration of immune and clinical data revealed different immunotypes related to poor clinical course *versus* improving health (36). Returning to our approach, it aims to identify distinctive transcriptional modules in the response of human leukocytes to SARS-CoV-2 infection in children and adults, and between oligosymptomatic and severe cases in both groups. The transcriptomic data thus obtained—distinctive transcriptional modules and their associated biological functions, highly connected and high significance genes, etc.—will be integrated with clinical and demographic data in order to gain a better understanding of the molecular mechanisms involved in the immune response to SARS-CoV-2 and, eventually, for identifying host-response predictors and potential therapeutic targets for drugs and vaccines.

## AUTHOR CONTRIBUTIONS

All the authors contributed equally to this study and have read and approved the final manuscript.
